# Integrating Care for Diabetes and Hypertension with HIV Care in Sub-Saharan Africa: A Scoping Review

**DOI:** 10.5334/ijic.5839

**Published:** 2022-01-31

**Authors:** Geoff McCombe, Jayleigh Lim, Marie Claire Van Hout, Jeffrey V. Lazarus, Max Bachmann, Shabbar Jaffar, Anupam Garrib, Kaushik Ramaiya, Nelson K. Sewankambo, Sayoki Mfinanga, Walter Cullen

**Affiliations:** 1 University College Dublin, Dublin, IE; 2 Liverpool John Moores University, Liverpool, UK; 3 Barcelona Institute for Global Health (ISGlobal), Hospital Clínic, University of Barcelona, Barcelona, ES; 4 University of East Anglia, Norwich, UK; 5 Liverpool School of Tropical Medicine, Liverpool, UK; 6 Shree Hindu Mandal Hospital, Dar es Salaam, TZ; 7 Makerere University, Kampala, UG; 8 National Institute for Medical Research, Dar es Salaam, TZ

**Keywords:** diabetes, AIDS, HIV, hypertension, review

## Abstract

**Introduction:**

Although HIV continues to have a high prevalence among adults in sub-Saharan Africa (SSA), the burden of noncommunicable diseases (NCD) such as diabetes and hypertension is increasing rapidly. There is an urgent need to expand the capacity of healthcare systems in SSA to provide NCD services and scale up existing chronic care management pathways. A scoping review mapped extant policy and evidence based literature on the feasibility of integrating NCD care with HIV in the region.

**Methods:**

A scoping review methodology was utilised to conduct a systematic search of peer-reviewed and grey literature published in English language and with no date limitation. A systematic search was conducted on PubMed, Embase, CINAHL, and the Cochrane library. The initial search identified 231 records considered for inclusion in this review. Twelve duplicate records were removed. The remaining 219 records were screened by title and abstract of which 165 records were excluded and 54 records were selected for full-text review. A further 16 records were excluded due to a lack of relevance or the unavailability of the full text article. Finally, 38 were charted and analysed thematically.

**Results:**

Thirty-eight studies were included. These comprised a range of different models to integrate NCD and HIV care in the region, reflecting differences in health system environments, and disease epidemiology. The studies provide a variety of evidence that integration of HIV and NCD care can be feasible and can improve clinical effectiveness and identify barriers and facilitators to integration and task shifting. The review confirms that integrated HIV and NCD care services is by-and-large feasible, being both clinically effective and cost-effective.

**Conclusion:**

The review may inform the understanding of how best to develop an integrated model of care service by reducing barriers to uptake, linkage and retention in HIV, diabetes and hypertension treatment in SSA countries.

## Introduction

There is an increasing noncommunicable disease (NCD) burden globally, with an estimated 1 billion people living with hypertension and about 9.4 million NCD-related deaths annually [1]. NCDs are important contributors to the burden of disease in countries at all stages of economic development, posing a high priority threat to public health worldwide [2,3]. Over three-quarters of global NCD deaths (28 million) and the majority of premature deaths (82%) occur in low- and middle-income countries (LMICs) [4]. Among global NCDs, type 2 diabetes mellitus (DM) is especially common [5,6]. The International Diabetes Federation recently reported that the incidence of DM would increase from 415 million in 2015 to 642 million by 2040, with more than 70% of the cases in LMICs [7].

Although HIV-infection is a leading cause of premature death among adults in sub-Saharan Africa (SSA), global NCD trends are mirrored in the region. The trend is evident from the rapidly increasing burden of NCDs such as DM and hypertension in SSA countries, giving rise to a dual HIV-NCD epidemic [8]. The Global Status Report on NCDs emphasizes that the negative impacts of NCDs are particularly severe in poor and vulnerable populations such as those living in the SSA region [8], where poverty exacerbates many health conditions [9]. In SSA, the prevalence of hypertension is increasing with 78% of adults over 55 years living with hypertension [10] and prevalence of DM is anticipated to double between 2010 and 2030; with 28 million people in SSA predicted to be living with the disease [11].

Since 2003, significant global investment has facilitated the establishment of HIV care services in the SSA region. The expansion of and improvements in life-saving antiretroviral therapy (ART) has decreased HIV related morbidity and mortality, leading to an ageing population living with HIV who are more susceptible to common NCDs such as hypertension. As such, management approaches in the region have transitioned from acute and emergency care to chronic care, making HIV programmes large-scale chronic disease initiatives. However, prevention, care, and treatment services for DM and hypertension remain inaccessible for most in SSA, and health systems are rarely designed to provide the continuing services required to effectively identify patients at risk, engage them in care, and retain them for long-term treatment [12]. Recent research indicates that only 5-20% of people with DM or hypertension are thought to be in regular care [13]. Therefore, there is an urgent need to expand the capacity of healthcare systems in SSA to provide services for NCDs such as DM and hypertension. As populations are increasingly demonstrating multi-morbidity, such as DM and hypertension with HIV [14], health care providers in SSA countries are now faced with an increasing need to manage HIV and NCDs simultaneously.

In the SSA region, as concern about the management of NCDs among people living with HIV (PLHIV) increases, the infrastructure and lessons learnt from the HIV chronic disease treatment model are important resources for a potential plan to expand and operationalise enhanced NCD prevention, care, and treatment [15]. These include health services which are stand-alone and vertically delivered and have been combined with decentralisation and task shifting, allowing primary health centres to treat large numbers of patients with almost 70% of people living with HIV-infection in regular care [16]. Given the similarities between chronic communicable and NCDs from the health system and program management perspective (their effects on health and individual functioning share common pathways and outcomes), the health care systems, tools to diagnose and manage patients, and implementation strategies developed to provide continuity of care for HIV in SSA can potentially be rapidly, efficiently, and effectively utilized to support patients and services for other chronic NCDs [15]. These include decentralised care, task-shifting, counselling, community engagement, drugs and diagnostics procurement and treatment adherence support for the management of HIV as a chronic condition [17,18]. However, though a number of models of integrated HIV/NCD care have been established in recent years, the lack of evidence-based care models for scaling up makes it difficult for SSA countries to develop effective policy and implementation of appropriate integration strategies [19].

We conducted a scoping review to map and describe extant policy and evidence based literature in the field, examine the feasibility of integrating care for DM and hypertension with HIV care in SSA, and examine how best to leverage infrastructure currently in place for HIV care, for the treatment of DM and hypertension. The review is part of the ‘INTE-AFRICA’ European Union (EU) funded project [20,21] which is assessing the effectiveness and feasibility of two approaches in Tanzania and Uganda: scale up hypertension and diabetes integrated services alone, or in combination with HIV infection services.

## Methods

A methodologically rigorous scoping review framework comprising an iterative six-stage process developed by Arksey and O’Malley [22] was adopted to undertake a comprehensive search of extant policy and evidence based literature examining the integration of NCD care with HIV care in SSA. The six stages of the scoping review process are described below.

### Stage 1: Identifying the research question

The objectives of this review were focused on extant knowledge and gaps around the content, evaluation, and effectiveness of integrating DM, hypertension and HIV care in SSA. Three research questions were developed to guide the review:

1. What is the existing policy and evidence based literature regarding the integration of DM and hypertension with HIV care in SSA?
2. Is it feasible to integrate care for DM and hypertension with HIV care in SSA?
3. How can we best leverage HIV care infrastructure for the treatment of DM and hypertension in SSA?

### Stage 2: Identifying relevant studies

The comprehensive three-step search strategy recommended in standard Joanna Briggs Institute (JBI) systematic reviews [23] was utilized in this review in order to identify both published and unpublished (grey literature) evidence. The first step was an initial limited search of relevant databases such as PubMed, followed by an analysis of the text words contained in the title and abstract, and of the index terms used to describe the article. A second search using all identified keywords and index terms was then undertaken across all included databases. Thirdly, the reference list of all identified reports and articles were manually searched for additional relevant studies. Only studies published in English were considered for inclusion in this review. No date limitation was imposed upon the search strategies.

**Figure 1:**
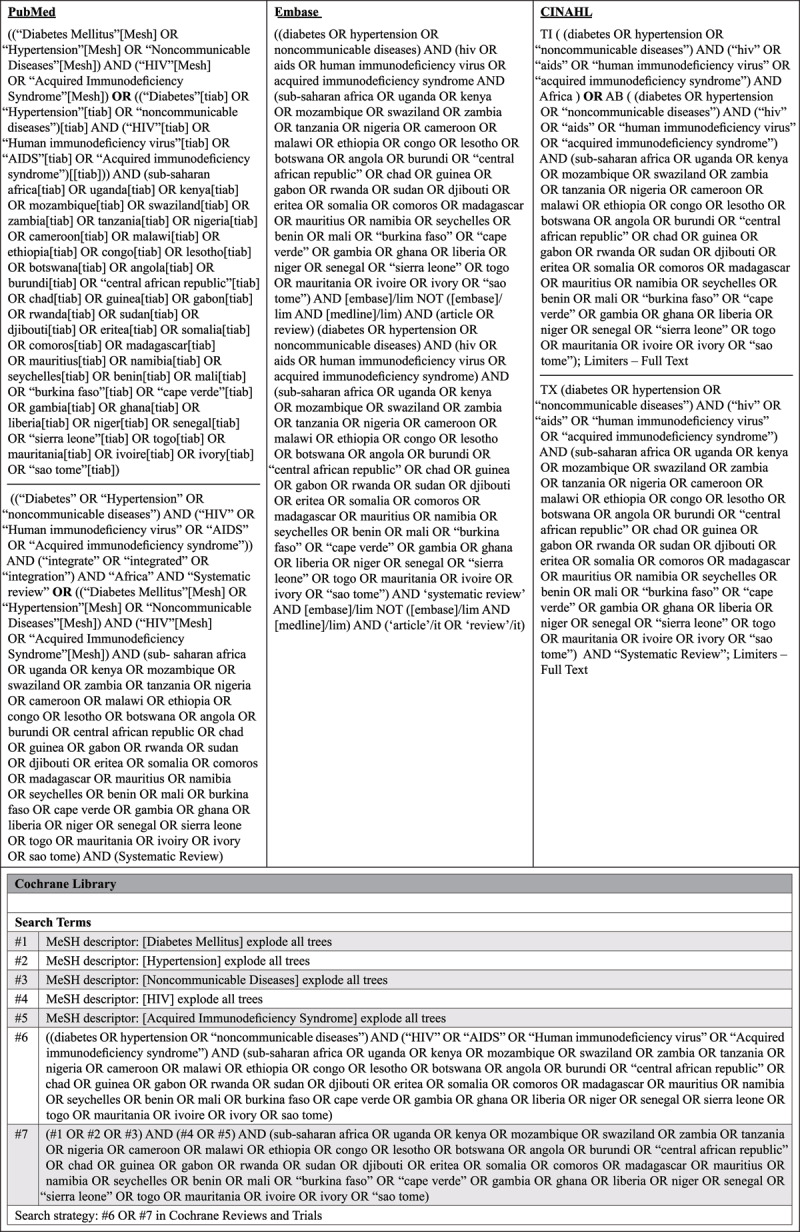
Search strategy.

Electronic databases searched included: PubMed, Embase, CINAHL, and the Cochrane library. Search terms agreed upon, and the general search strategy are outlined in ***Figure 1***. Citations were managed using the bibliographic software manager EndNote, with duplicates removed manually.

### Stage 3: Study selection

**Figure 2:**
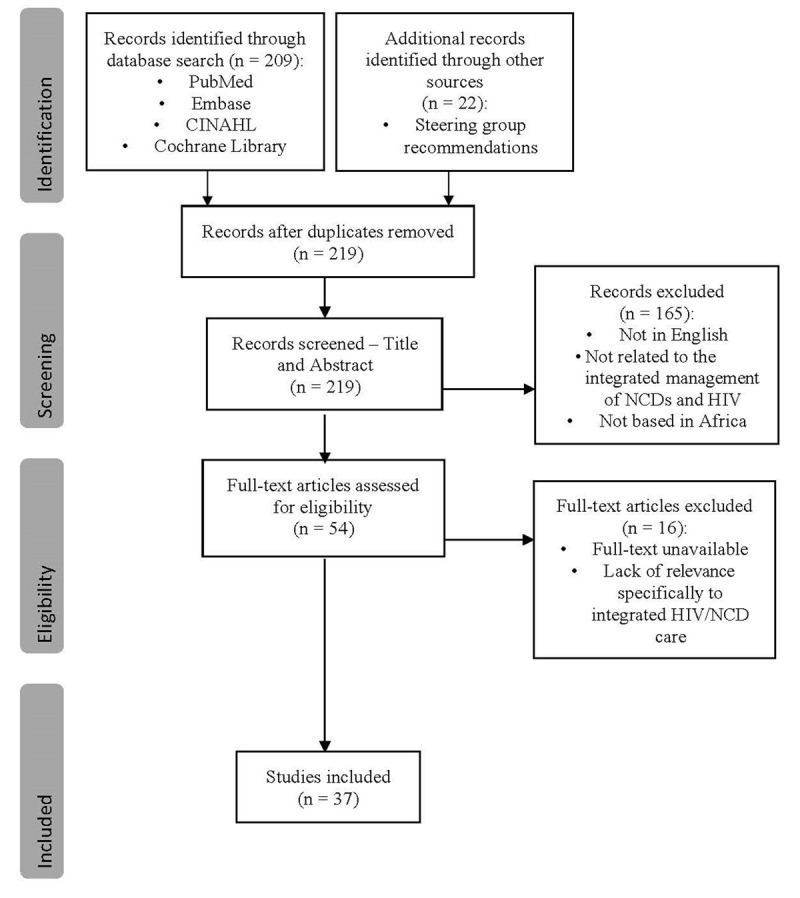
PRISMA flow-diagram of the study selection process of identification, screening, and inclusion criteria.

The selection process consisted of two levels of screening: (1) a title and abstract review and (2) a full-text review. In the first level of screening, the titles and abstracts of identified articles were screened for inclusion against a set of minimum inclusion criteria (abstract relevant to the topic area). In the second screening, the full text of articles was assessed to determine if they meet the inclusion criteria (full text is relevant to the study title). The PRISMA flow diagram as illustrated in ***Figure 2*** outlines the results of the literature search.

Consistent with the scoping review methodology, this study was broad in its inclusion of different types of literature [22,24,25]. and an assessment of methodological quality was not performed to include or exclude studies based on quality scores [22,24,26]. Both peer-reviewed and grey literature were searched, with no methodological requirement for study inclusion. This facilitated the inclusion of an array of literature, which included quantitative, qualitative and mixed-method studies, as well as systematic reviews and meta-analyses. Protocols were excluded as they did not provide evidence required, but where relevant and available, studies published based on the protocols identified were screened for inclusion. The eligibility criteria for content were developed according to the JBI reviewer’s manual (2015) [27], which suggests the use of the mnemonic PCC (*population, concept*, and *context*) to target the desired focus and scope for the review (***Table 1***) and the following pre-determined exclusion criteria. All criteria had to be met for inclusion/exclusion in the final review.

- Not in English
- Not related to the integrated management of NCDs and HIV
- Not based in sub-Saharan Africa

**Table 1:** PCC Framework.


CRITERIA	DETERMINANTS

Population	The article had to focus on individuals with NCDs (DM or hypertension) and/or HIV, without restrictions on age or sex.

Concept	The article had to describe, evaluate, or propose how NCD management can be integrated with HIV management to improve patient outcomes.

Context	The article had to focus on the integration of NCD management with HIV management in the context of SSA.


### Stage 4: Charting the data

To facilitate comparison and thematic analysis the following data were extracted from the articles:

- Author(s), year of publication, title
- Study setting
- Study design
- Target population and diseases
- Intervention type
- Outcomes measured
- Findings

### Stage 5: Charting, summarizing and reporting the results

A thematic analysis was conducted of the charted articles for inclusion in the final review using the World Health Organisation (WHO) definition of integrated service delivery [28]. The initial thematic analysis was completed by the first author (GM) using Braun and Clarke’s framework [29]. Steps 4 and 5 of the framework were then carried out together by the first, second and last author.

Two tables were developed to summarize findings from the included studies (***Table 2 (Appendix 1)*** and ***Table 3*** below). The tables enable better contextualization of what is known from the existing literature about how we can best integrate DM and hypertension management with HIV management in SSA.

**Table 3:** Descriptive characteristics of final included studies (Intervention, Outcomes Measured, Findings).


AUTHOR, YEAR	DESCRIPTION OF INTERVENTION	OUTCOMES MEASURED	FINDINGS

Anand et al., 2019 [30]	Task-sharing: sharing of tasks with other healthcare professionals with some supervision/referral to physicians	Population, interventions, blood pressure, and task sharing groups	(Task sharing in LMICs has been useful in managing HIV/AIDS) Task-sharing interventions for managing hypertension in LMICs show potential in reducing blood pressure

Chang et al., 2019 [31]	Integrated chronic disease management model for managing multimorbidity	Relationship between type of multimorbidity and progression along the care continuum; Effect of type of multi-morbidity on HIV care among HIV patients	Among HIV patients, presence of cardiometabolic conditions was associated with less progress in HIV care. The findings imply that the objective of the ICDM model may not yet be realised

George et al., 2019 [32]	n/a	Association between detectable HIV viral load and NCD comorbidity	There is an underdiagnosis of NCDs in HIV patients. An integrated chronic care system would allow enhanced detection, and dual management of HIV and NCDs

Iwelunmor et al., 2019 [33]	Integrating evidence-based hypertension interventions within HIV clinics for PLHIV in Nigeria	Capabilities, Opportunities and Motivations for integrating evidence-based hypertension interventions within HIV clinics for PLHIV in Nigeria	Relative capabilities and opportunities were minimal. There is a need to strengthen the HIV clinics in Lagos for the implementation of these interventions to improve patient outcomes and service delivery in Southwest Nigeria

Kwarisiima et al., 2019 [34]	Integrated chronic care delivery model at local health facilities that offered treatment for both HIV and hypertension	Primary: Hypertension control at follow-up clinic visits Secondary: Blood pressure control at 2 consecutive visits separated by at least 1 month	Integrated HIV and hypertension care provided under the same roof enabled “one-stop” shopping for patients. As hypertension and chronic care for other NCDs is integrated with HIV chronic care across SSA, co-located services, a well-trained workforce, and clinic infrastructure will likely be crucial to successful treatment of both

Ojo et al., 2019 [35]	Integrated CVD-HIV care	Concept, taxonomy and alignment of feasibility; Specific metrics used in feasibility assessment	Several feasible interventions that integrated multilevel CVD and HIV care were identified: Multi-disease screening; Referral and linkage to care. Treatment assessment of feasibility was not conducted in a consistent fashion across studies

Ansbro et al, 2018 [36]	Staff training; locally adapted protocols; chronic care files; a revised appointment system and patient flow; and a new database	Program processes, effectiveness and costs	NCD care can be integrated into a HIV department and outpatient setting in an MSF-supported primary care centre by utilising pre-existing structures, and can achieve acceptable intermediate clinical outcomes and retention rates at a cost that is similar to HIV programs.

Ekrikpo et al., 2018 [37]	n/a	Prevalence and correlates of hypertension, DM, obesity and dyslipidaemia	High prevalence of CVD risk factors makes it imperative to ensure detailed screening for CVD in HIV patients at care initiation and at regular intervals during follow-up. An integrated approach to NCD/HIV care may be the answer to this double disease burden

Golovaty et al., 2018 [38]	Integrated home-based HIV–NCD testing and counselling conducted by lay counsellors trained by a study nurse in HIV testing, anthropometric measurement, and point-of-care NCD screening	Program micro-costing, no of persons tested per day	Comprehensive home-based HIV-NCD testing and counselling results in modest increase in costs with the potential to avert NCD death and disability

Haldane et al., 2018 [39]	Service integration for HIV/AIDS with NCDs	Outcomes of service integration, barriers and facilitators to integration	Several innovative initiatives have been described for integrating CVD, hypertension and diabetes services with HIV/AIDS services. These highlight the importance of using communities as a locus of action for activism, advocacy, accountability and service delivery and the importance of partnerships to facilitate multidisciplinary collaborations

Juma et al., 2018 [40]	NCD behavioural change communication (BCC) interventions	Reach, Effectiveness, Adoption, Implementation, and Maintenance	Although data on integrated HIV/NCD health promotion efforts is limited, evidence is found to support the feasibility of integrating HIV/NCD within multi-disease screening campaigns in Uganda, Kenya and South Africa

Matanje et al., 2018 [41]	Integrated HIV/NCD care	Availability of policies and programs and ongoing interventional processes related to integrated HIV/NCD care	Leveraging highly funded and successful HIV programs in SSA that employ proven monitoring and evaluation systems will support effective HIV/NCD integration program monitoring and provide an opportunity to improve equity and access of NCD care

Njuguna et al, 2018 [19]	Models of integrated HIV/NCD care	Challenges/barriers, facilitators/successors of integrated care models, lessons learnt	Leveraging existing HIV infrastructure to provide NCD care to the SSA population is feasible, with various approaches possible depending on available program capacity. Future efforts will need to factor in descriptions of process metrics and cost-effectiveness to further guide implementation, as well as clinical outcomes to aid decision makers.

Nuche-Berenguer and Kupfer, 2018 [42]	Integration of diabetes care into existing HIV and TB platforms	Diagnostic and treatment capacity for diabetes; Interventions aimed to increase capacity; Integration of diabetes care into existing HIV platforms	Many challenges remain for chronic diabetes care provision. Optimizing existing resources by integrating diabetes care with other disease platforms e.g. HIV and TB care is a great opportunity to improve diabetes diagnostic systems, medicines provision, training of health personnel, patient empowerment, and tracking of disease burden

Patel et al., 2018 [43]	NCD-HIV integrated programs with screening and management approaches that are contextually appropriate for resource-limited settings	Burden of NCDs among PLHIV in LMICs; Current management of the diseases	Improved data collection and surveillance of NCDs among PLHIV in LMICs are necessary to inform integrated HIV/NCD care models. Although efforts to integrate care exist, further research is needed to optimize the efficacy of these programs

Pfaff et al., 2018 [44]	Integrated HIV-NCD care: blood pressure measured at every visit and random blood glucose determined every 2y in a large HIV clinic	Patients screened and diagnosed with hypertension/diabetes, mean duration of ART visits, mean number of patients seen a day, challenges of integrated HIV-NCD care	The experience in Zomba demonstrates that the strengths of the ART program may be used to provide integrated NCD care within HIV settings. Several challenges related to workload, patient flow, monitoring and evaluation and NCD drug shortages were encountered during pilot integration implementation that are currently being addressed and may serve as lessons for wider integration efforts

Rawat et al., 2018 [45]	Integration of HIV care into primary care clinics	Influence of integrating HIV-care into primary health care clinics (PHCCs) on quality of NCD care for diabetes and hypertension; how changes may relate to HIV patient numbers	The gains in infrastructure and investment in HIV-care could be leveraged to strengthen, not erode, NCD care. By harmonizing preventive efforts to reduce and treat NCDs within the context of HIV-care, countries can synergistically advance health and social benchmarks. The growing burden of other NCDs combined with the greater life expectancy of people living with HIV demand that health systems remain strong to ensure that comprehensive HIV-care does not come at the expense of screening and treatment for NCDs, especially in PHC settings

Ameh et al, 2017 [46]	Integrated chronic disease management (ICDM) model in Primary health care facilities	Health outcomes including patients’ CD4 counts and blood pressure	Application of the model had a small effect in controlling patients’ CD4 counts and BP, but showed no overall clinical benefit for the patients; hence, the need to more extensively leverage the HIV program for hypertension treatment

Mosha et al., 2017 [47]	Integrating blood pressure screening into a community-based HIV screening program	Prevalence of hypertension and the associated factors	Integration of hypertension screening into routine clinics for HIV allowed many undiagnosed cases of diabetes and other chronic diseases to be identified. If combined with proper hypertension treatment, consequences such as CVD and stroke can be avoided, but this requires improvements of the clinical services in health facilities

Pfaff et al., 2017 [48]	NCD care at EQUIP-affiliated facilities (Extending Quality Improvement for HIV/AIDS in Malawi)	Current capacity of NCD program, using hypertension and diabetes as tracer conditions; Extent of integration with HIV and ART care	There is much potential to use lessons from the ART program to strengthen NCD care as ART clinics represent one of the first screening and primary care delivery models for chronic disease in Africa, supported by health information, short course training and supervision

Shankalala et al., 2017 [49]	n/a	Proportion with impaired fasting glucose or DM	High levels of impaired fasting glucose or DM among cART patients compared to what is reported, suggesting missed care and support opportunities. There is a need to repackage HIV programming to include integration of diabetes screening as part of the overall care and support strategy

Divala et al, 2016	n/a	Proteinuria, non-fasting lipids and cardio/cerebro-vascular disease (CVD) risk scores	Integrated HIV-hypertension-diabetes care may be individually beneficial but increases the burden of care for busy HIV clinics. Excluding patients with mild hypertension and low CVD risk from drug treatment would half the overall burden of HIV patients in need of integrated pharmacotherapy for diabetes and/or hypertension

Fairall et al., 2016 [50]	Primary Care 101 (PC101) leveraging health system reforms accompanying the scale-up of antiretroviral therapy (ART) to improve quality of primary care for other priority conditions e.g. NCDs	Treatment intensification for hypertension, diabetes, and chronic respiratory disease; case detection of depression	Educational outreach to primary care nurses to train them in the use of a management tool involving an expanded role in managing NCDs was feasible and safe but was not associated with treatment intensification or improved case detection for index diseases. However, the intervention, with adjustments to improve its effectiveness, has been adopted for implementation in primary care clinics throughout South Africa

Rachlis et al., 2016 [51]	Chronic disease care for HIV, TB and hypertension as part of the Academic Model Providing Access to Healthcare (AMPATH) program	Barriers and facilitators to linkage and retention	Integrated service delivery for chronic diseases may support timely engagement in care given the increasing prevalence of NCDs among the general population and HIV patients. However, such models should be rigorously evaluated to explore their acceptability and potential negative impacts, and should be tailored for specific contexts and settings, and support individuals with logistical and financial challenges

Some et al., 2016 [52]	Task-shifting the management of NCDs from clinical officers to nurses who also manage routine primary and HIV care	Adherence to Médecins Sans Frontières clinical protocols	Nurses working within a resource-constrained, primary care and HIV setting, can successfully follow protocols managing stable patients with multiple NCDs. This approach could be applied in other similar HIV-based programs to extend access to areas with increasing need of NCD care and limited resources

Edwards et al, 2015 [53]	n/a	Patient demographics, clinical characteristics, disease prevalence	PLHIV are at higher risk of developing concurrent NCDs at a younger age and would benefit from routine screening and treatment. Treatment appears to produce results comparable to patients without HIV. It also demonstrates that it is possible to integrate both HIV and NCD care together in a primary care program that is largely run by clinical

			officers and nursing staff within significant resource constraints. This model may be useful in the scale-up of NCD care in sub-Saharan Africa in the future

Haregu et al., 2015 [54]	HIV-NCD integration	Rationale, policy bases and models of HIV-NCD integration	Models of HIV-NCD integration that were “tested” in the context of developing countries vary but all models indicated that the integrated approach was feasible, effective, efficient and acceptable. However, overall evidence is limited and context-specific evidence is lacking

Khabala et al, 2015 [55]	Combined MACs: Nurse-facilitated groups of 25-35 stable hypertension, diabetes and HIV patients with quarterly follow-ups	Correctly completed blood pressure, weight and laboratory testing during MAC attendance, adherence, referral to clinic, retention	This study demonstrates the feasibility and early efficacy of Medication Adherence Clubs as a novel group treatment model to care for stable patients with mixed chronic diseases in an urban, resource-constrained, informal settlement. It supports reducing the burden of regular clinical follow-up among stable patients and improves the flexibility of care delivery

Mahomed and Asmall, 2015 [56]	Integrated chronic disease management (ICDM) model using a health systems approach	Feasibility of integration, Challenges	The implementation of the integrated chronic disease management model is feasible at primary care in South Africa provided that systemic challenges and change management are addressed during the implementation process

Joshi et al., 2014 [57]	Task-shifting the management of NCDs from physicians to non-physician healthcare workers (NPHWs)	Viability, Cost-effectiveness, Clinical effectiveness	Task shifting has proved to be a viable and cost-effective option for the management of HIV-AIDS in SSA. Task-shifting, if accompanied by health system restructuring, is a viable and potentially effective and affordable strategy for the management of NCDs

Govindasamy et al, 2013 [58]	n/a	Yield of new diagnosis, CD4 count testing, linkage to care, correlates of linkage and barriers to care	Integrated HIV, TB symptoms and NCD screening in mobile units holds promise for expanding the scope of HIV services and the reach of primary healthcare

Chamie et al, 2012 [59]	A five-day, multi-disease campaign, offering diagnostic, preventive, treatment and referral services Community-based HIV and NCD testing campaigns offering diagnostic, preventive, treatment and referral services compared with routine service delivery methods	Community participation, case-finding yield, and linkage to care Feasibility and diagnostic yield of integrating NCD into a rapid community-based multidisease screening campaign	In an integrated campaign engaging 74% of adult residents, a high burden of undiagnosed HIV, hypertension and diabetes was identified. The campaign demonstrates the feasibility of integrating hypertension, diabetes and communicable diseases into HIV initiatives

Rabkin et al., 2012 [18]	Intervention package adapted and implemented from HIV program to support diabetes services in the outpatient department.	Site assessments, chart review, and health care worker questionnaires.	Countries which have successfully scaled up HIV services have already learnt profound lessons about chronic care delivery. Using locally owned and contextually appropriate resources may be an efficient and effective way to “jumpstart” NCD programs and to strengthen health systems

Van Olmen et al., 2012 [60]	Models of care delivery and treatment of diabetes and HIV/AIDS	The disease dimension,” the “health provider dimension,” the patient or “person dimension,” and the “environment dimension” of chronic diseases.	Lessons from present care models for HIV/AIDs and DM show potential for cross-fertilization between models: rapid scale-up approaches through the public health approach by simplification and decentralisation; community involvement, peer support and self-management strategies; and strengthening health services

Levitt et al., 2011 [61]	Integration of primary level care for people with NCDs and those receiving ART	Health care implications of colliding HIV/NCD epidemics, Argument for an integrated model of care	The current vertical program for HIV/AIDS care and that for NCDs, which take place within a chaotic primary care system, should be replaced by integrated care for patients with any chronic disease, in a coherent patient-centred program Integrated chronic disease care services in primary care facilities should be separated from acute care services, naturally with sufficient coordination between the two forms of care to ensure that patients can move freely between acute and chronic care services when required

Lekoubou et al., 2010 [62]	Task shifting to nurses: Nurse-led strategies for chronic disease management	Surrogates of disease control	Task sharing in LMICs has been useful in managing HIV/AIDS. Task-shifting has been implemented in a few countries in SSA with some indicators of success in chronic disease care. With regard to hypertension and diabetes, limited available evidence suggests that acceptably designed task-shifting interventions are feasible and useful for their care in SSA

Maher et al., 2010 [63]	Primary care-delivery for both CDs and NCDs	Ways primary care can respond to the health transition to a double burden of CDs and NCDs in Africa	Primary care is well placed to offer a coherent response to the problems of both CDs and NCDs. National and international alliances have a key role to play in mobilizing necessary investments for an effective primary-care response to the health transition in Africa.


* PLHIV – People living with HIV. * LMIC – Low-and-middle income countries.

### Stage 6: Consultation with stakeholders

The 209 records identified in the initial database search were shared with members of the ‘INTE-AFRICA’ consortium [20,21] which resulted in an additional 22 records being suggested.

### Patient and Public Involvement

As this was a scoping review it was not appropriate to involve patients or the public in the study.

## Results

### Search Results

The initial database search identified 209 records. These, along with an additional 22 records suggested by the steering group which were not identified in the initial database search, gave a total of 231 records which were considered for inclusion in this review. After 12 duplicate records were removed, reviewers screened the remaining 219 records by title and abstract, during which 165 records were excluded for reasons showed in ***Figure 2***. Fifty-four articles met the inclusion criteria and were selected for full-text review. Following full-text review, 16 records were excluded due to a lack of relevance or the unavailability of the full text article (***Figure 2***), leaving 37 records which were identified to be relevant to the integration of NCDs management with HIV management in Africa. The search process, as guided by the Preferred Reporting Items for Systematic Reviews and Meta-Analyses (PRISMA), is summarised in ***Figure 2***. Data were extracted from a final selection consisting of 37 records which met the eligibility criteria for the review.

### Description of Included Studies

The 37 records included examined various aspects related to the integration of HIV and NCD care in SSA. The records included studies that examined the feasibility of integrating care for DM and hypertension with HIV care, as well as studies that examined how best to leverage the infrastructure and lessons learnt from providing HIV care to enhance the treatment of DM and hypertension. Both quantitative and qualitative studies were included, with no restriction on study design, to allow us to map the field and aquirea sense of the extant literature available on the topic of HIV/NCD care integration in SSA. Papers included were published between 2010 and 2019, and study locations included Kenya, South Africa, Swaziland, Tanzania, and Uganda. General characteristics of the included studies are summarized in ***Table 2 (Appendix 1)*** and ***Table 3***.

### Mapping the Field – Extent and Nature of Extant Literature

Despite no limitations being placed on publication date in our database searches, the earliest included record was published in 2010. This is understandable, given that integrated HIV/NCD management remains a relatively new area of interest, with the emerging NCD epidemic in SSA only more recently being put under the national and global spotlight, in contrast to the longstanding HIV epidemic that has been recognized for over three decades.

With no limitations being placed on the study design of records included in this review, the included records adopted a wide range of methodologies. Of the 37 records, 13 were observational studies, including cross-sectional studies, case-control studies, as well as retrospective and prospective cohort studies. Eight were interventional studies, including pre-post studies, non-randomized trials, and randomized controlled trials. Two were mixed methods studies, two were qualitative studies, and 13 were reviews, including systematic reviews, scoping reviews and narrative reviews. Study designs of included records are summarised in ***Table 2 (Appendix 1)*** and ***Table 3***.

### Integrating Findings

Studies included identified various aspects of integrating NCD care into HIV care, which can be broadly classified into three themes: (1) Evaluation of HIV/NCD Care Integration, (2) Arguments for Integrated HIV/NCD Care, and (3) Effectiveness of Task-Shifting/Sharing for NCD Care Delivery. The themes explored by the included records are summarized in ***Table 2 (Appendix 1)***.

#### Evaluation of HIV/NCD Care Integration

Of the 37 records, 24 evaluated various aspects of HIV/NCD Care Integration. These can be further classified into three sub-themes: ‘Current Policies and Integration Models: Summary/Review’, ‘Feasibility: Capacity, Clinical- and Cost-effectiveness’, and ‘Facilitators and Barriers to Integration’. These sub-themes are not mutually exclusive, with some records falling under two or indeed all of the three subthemes.

### Current Policies and Integration Models Summary/Review

Seven of the 24 records under this theme summarized and reviewed various policies and models related to integrated HIV/NCD management that are currently in place in various African countries. Of these seven studies, one was an observational study [41], one was a mixed- methods study [48], one was a qualitative study [54], and four were review papers [19,39,40,42]. The seven records reported studies in various SSA countries, including Kenya, Malawi, South Africa and Swaziland. Two of the seven records summarized and reviewed current policies in place in SSA countries for HIV/NCD integration [41,54]. These studies highlighted National stakeholders were supportive of integrated policy development, however inconsistencies in implementation where evident between countries. Evidence gaps for cost-effectiveness, effects of integration on key HIV and NCD outcomes and funding mechanisms for sustained implementation of integrated HIV/NCD care strategies, were among challenges identified. All seven records summarized and reviewed current HIV/NCD care integration models in SSA countries [19,39,40,41,42,48,54]. These included integrated community-based screening for HIV and NCDs in the general population; screening for NCDs and NCD risk factors among HIV patients enrolled in care; integration of HIV and NCD care within clinics; differentiated care for patients with HIV and/or NCDs; and population healthcare for all. The studies acknowledged integration models are in their infancy, resulting in a lack of clinical and process outcomes data, and a lack of cost-effectiveness data from the various models.

### Feasibility: Capacity, Clinical- and Cost-effectiveness

All 24 records under this theme evaluated the feasibility of integrated HIV/NCD management in SSA countries. These records discussed aspects such as the capacity of current HIV clinics to provide integrated care, as well as clinical- and cost-effectiveness of integrated care. Of these 24 studies, seven were observational studies [31,36,38,41,47,53,64], seven were interventional studies [18,34,44,45,46,56,59], two were mixed methods studies [33,48], two were qualitative studies [51,54], and six were review papers [19,35,39,40,42,43].

Four of the 24 records examined the capacity of HIV/ART clinics to accommodate integrated NCD care into care delivery [33,41,42,48]. Studies indicated that HIV/ART programmes have developed considerable systems innovations which could accommodate NCD care. Nineteen records assessed the clinical effectiveness of integrated HIV/NCD care. For the purposes of this study, studies focusing on patient-centred outcomes such as: population penetrance [35,40], disease control as measured using surrogate disease markers [34,35,36,39,42,46,53], linkage and retention in care [19,35,36,42,51,54,59,64], quality of care (wait-time, service delivery, comprehensiveness and continuity of care etc.) and patient satisfaction [18,19,31,39,40,42,44,54,56], treatment adherence [40,42,54], and diagnostic yield [19,35,39,40,42,45,47,53,59,64] are classified as studies assessing “clinical effectiveness”. Most of the studies concluded in favour of the clinical effectiveness of integrated HIV/NCD care in view of improvements that were achieved in the above-mentioned patient-centred outcomes. These included screening for NCDs within HIV care programmes which improve the identification of undiagnosed NCDs among patients living with HIV and a reduction in duplication and fragmentation of services, which increase efficiency of resource use and help patients remain in care. Five records examined the cost-effectiveness of integrated HIV/NCD care [34,36,38,40,59]. These studies support the cost-effectiveness of HIV/NCD integration efforts while highlighting the additional time burden of NCD screening and testing along with human resources and drugs prices as the major driver of costs.

### Facilitators and Barriers to Integration

Seventeen of the 24 records under this theme identified various facilitators and barriers to integrating or to achieving efficient integrated HIV/NCD care (see ***Table 4***). Of these 17 records, four were observational studies [31,36,41,53], five were interventional studies [18,44,45,46,56], two were mixed methods studies [33,48], and six were review papers [19,35,39,40,42,43].

**Table 4:** Barriers and facilitators to integrated HIV/NCD care.


BARRIERS	FACILITATORS

The lack of diagnostic equipment and medication [18,19,31,35,36,39,42,43,44,45,46,48,53,56]	Task-shifting/task-sharing [19,42,43]

Lack of trained staff or training [18,31,35,36,39,41,42,43,46,48,53,56]	Utilization of existing trained staff or infrastructure [19,31,35,39,40,42,45,48]

Overburden from an increased workload [19,36,39,40,43,44,45,46,48,56]	The perceived benefits of integrated care [35,43,44,48]

Evidence gaps [40,41,42,43]	Favourable policy environment and governmental support [19,35,41]

Insufficient funding [19,35,40,41,48]	Reorganization of patient flow [19,44]

Lack of guidelines and operating protocols [18,53]	

Perceived threat of integration to existing HIV success [40,41]	


#### Arguments for Integrated HIV/NCD Care

Eight of the 37 included records put forth arguments for integrated HIV/NCD care. Of these eight records, four were observational studies [32,37,49,65], and four were review papers [19,60,61,66]. The four observational studies [32,37,49,65] and one review paper [60] examined the prevalence of either NCDs [32,49,65], CVD risk factors [37,65] in PLHIV in SSA countries such as Malawi, Nigeria, South Africa and Zambia. All five studies found a high prevalence of these conditions in PLHIV, and proposed integrated care as a solution to provide holistic chronic care, enhance detection and management of the diseases, enhance NCD prevention, as well as to reduce cost and increase efficiency in a resource-limited setting. The three remaining review papers considered the consequences of the rising dual epidemics on the organization of care and potential bridges between the care of these diseases [60,61,66]. Overall, studies indicated HIV/NCD care integration is a feasible approach to health system strengthening in SSA but must be context specific. Njuguna et al. used case studies to demonstrate a variety of HIV/NCD integration models which programs can adopt and adapt based on available resources, needs and priorities. These included the creation of curricula to produce integrated chronic care providers instead of specific staff dedicated only to HIV or NCD roles and decentralisation to bring care closer to patients to minimize logistical barriers to access, while reducing congestion at ‘central’ health facilities.

#### Effectiveness of Task-Shifting/Sharing for NCD Care Delivery

Six of the 37 included records evaluated the effectiveness of various task-shifting/task-sharing interventions for NCD care delivery. Of these six records, two were observational studies [52,55], one was an interventional study [50], and three were review papers [30,57,62]. Task-shifting is defined as the rational movement of primary care duties from physicians to non-physician health-care workers (NPHWs), whereas task-sharing is a planned strategy in which a team of health-care professionals work together to deliver a service, accompanied by training or certification and support for health-care workers [30]. These strategies have been employed in developing countries, in particular in the SSA region, with compelling evidence on feasibility and indicators for success for various disease entities and health sectors including HIV, TB, mother and child health [62], and to a lesser extent, NCDs [52], and have been recommended by WHO for low and middle income countries, due to their low health care worker to patient ratio [52].

## Discussion

### Summary of key findings

The review sought to answer three research questions:

*1. What is the existing policy and evidence based literature regarding the integration of DM and hypertension with HIV care in SSA?*

Several studies identified different models of HIV-NCD service integration [19,60,61]. These include NCD prevention and control incorporated into existing HIV services; HIV prevention and control added to primary healthcare already providing NCD services, and simultaneous introduction of integrated HIV and NCD services. In terms of existing policy, studies show that National stakeholders, led by Ministries of Health (MOH), are supporting HIV/NCD integration policy development and implementation through evidence generation and coordination activities [41,54]. However, the degree to which the push for HIV/NCD integration is reflected in national level policy documents varies geographically.

2. *Is it feasible to integrate care for DM and hypertension with HIV care in SSA?*

The review indicates that integrated HIV/NCD care in the SSA region is challenging but potentially feasible. However, studies highlight there remain various country specific, contextual, human resource, logistical and infrastructural barriers that need to be considered in order to best achieve efficient and effective integrated care delivery [33,35,36]. One way of overcoming the manpower and infrastructural strain due to the acute shortage of trained health staff in SSA, would be through task-shifting, which has proven feasible as an alternative to rapidly increase the health workforce [30]. This will require enhanced focus on NCD training cascade in integrated services. From the studies included in this review it is not possible to reach a definitive conclusion on whether it is feasible to integrate care for DM and hypertension with HIV care in SSA. Further research is required to generate evidence on the benefits, challenges and cost-effectiveness of integration of HIV and NCD services, particularly in those countries where NCD services are still developing.

3. *How can we best leverage HIV care infrastructure for the treatment of DM and hypertension in SSA?*

While the review indicates multiple ways in which to leverage the lessons and resources of HIV programs when working to expand and enhance DM and NCD services in SSA, it also emphasises no single approach is likely to work in all settings. In some countries, integration of chronic disease services for HIV and NCDs at the point of care may be an effective approach [46]. In other settings this may be neither feasible nor desirable. Studies suggest that countries which have successfully scaled up HIV services have already learned profound lessons about the delivery of chronic care. Using these locally owned and contextually appropriate resources may be an efficient and effective way to kickstart NCD programs and to strengthen health systems to support longitudinal services for all [18,41,65].

### How this review relates to other literature

Although this topic has been studied before [37,49,65], the best practices in integrated care provision for the prevention, identification and treatment of hypertension, diabetes and HIV in SSA have yet to be identified. While the potential benefits of HIV-NCD integration for the health system and patients are well documented [67,68], consistent with this review, research indicates differences in health systems preparedness to reconfigure the health system environment when it comes to chronic care delivery among the diverse SSA countries make implementing an integrated care model for HIV-NCD challenging [69]. The majority of models of HIV/NCD integration that have been trialled or implemented in SSA in this review and the wider literature have highlighted the need for integration programs to be context-specific, with no single approach likely to work in all settings [70].

Therefore, an enhanced contextual understanding of integration is further warranted. Service experience and patient outcomes are very dependent on understanding cultures and contexts surrounding integrated service design, and the inputs and actions of policy makers, healthcare providers, clinicians, patients, communities and international donors With these in mind, the policymakers at national and regional levels can then devise a contextually appropriate strategy that maximises existing resources and leverages upon the strengths of the local health system while preventing, and prevailing cracks from hurting hard-earned HIV gains and quality of clinical care.

Implications for research and policyWhile the quantity and quality of research on integrated HIV/NCD care are and have been steadily increasing over the past decade, there remains limited evidence about integration both in terms of scope and generalizability of the evidence [16]. Existing evidence is limited to small scale feasibility studies in largely different contexts, lacking large scale or randomized and/or controlled evaluations [71]. There is also a general lack of clinical and process outcomes data, and of cost-effectiveness data from the various models, which are often required prior to large-scale HIV/NCD intervention programs being rolled out. In addition, context-specific evidence needed to adapt an integrated care model that is best suited to the needs of a specific country is lacking. These evidence gaps are often cited as a barrier to integration in SSA countries, suggesting an urgent need to fill these gaps to further inform implementers and policymakers. Hence, further research is warranted to gather not just more, but higher quality evidence on outcomes and cost through randomized controlled trials, as well as country-specific evidence at operationalisation and scale up over time. Evidence on regional, national and subnational context-specific variations in burden of NCDs in PLHIV should inform policy decisions, priority-setting and resource allocation, while evidence on the impact of HIV/NCD integration on the existing HIV care model should inform policymakers on the likely effects of integration on HIV targets, indicators and systems. Efforts should further concentrate on both HIV and NCD awareness raising tactics and prevention activities. Additionally, further research is required to understand public understanding of the HIV/NCD burden of disease and integration of services, understand and respond to the scaleup process and determine the sustainability of integrated care models. It is the aim of the ‘INTE-AFRICA’ project [20,21] to address these evidence gaps, along with the various barriers to integration identified in this review by conducting a randomised control trial, health economic and process evaluation in the operationalisation and scale up of integrated clinics in Tanzania and Uganda

### Methodological considerations

Several limitations should be considered when interpreting the findings of this review. Whilst we adopted the rigorous scoping review methodology and used a comprehensive search approach, there is a possibility that not all publications relevant to the inclusion criteria were identified by the searches or databases used. Scoping reviews do not include an assessment of study quality as the focus is on covering the range of work that informs the topic rather than limiting the work to studies that meet particular standards of scientific rigour. Finally, only articles published in English were considered for inclusion in our review, which could have resulted in the exclusion of equally relevant literature published in other languages used in the SSA region such as Portuguese, French and local African languages.

## Conclusion

Our review illustrates that HIV/NCD care integration in sub-Saharan Africa is potentially feasible, with several models available that countries can adopt and adapt based on available resources, needs, and priorities. However, it is important to note that existing evidence is largely limited to small-scale feasibility studies in varying contexts, with a paucity of higher quality research measuring clinical outcomes, cost-effectiveness and the sustainability of such integration. Conducting high quality trials and implementing their findings will enable the optimization of existing resources and enhance the outcomes of NCD detection, treatment and care for all patients in a manner that is both cost-effective, and which does not weaken the well-functioning HIV efforts in the SSA region.

## Appendices

**Appendix 1:** Table 2. Descriptive characteristics of final included studies (Setting, Design, Population).

AUTHOR, YEAR	NAME OF STUDY	SETTING	STUDY DESIGN	TARGET POPULATION AND DISEASES

Anand et al., 2019 [30]	Task sharing with non-physician health-care workers for management of blood pressure in low-income and middle-income countries: a systematic review and meta-analysis	Low- and middle-income countries in Africa, Asia	Systematic review and meta-analysis	Intervention studies: trials (n = 43), before-and-after studies (n = 20) Hypertension, cardiovascular risk factors

Chang et al., 2019 [31]	Multimorbidity and care for hypertension, diabetes and HIV among older adults in rural South Africa	Agincourt, Mpumalanga, South Africa	Cross-sectional data analysis	Adults (n = 4,447) enrolled in the Health and Aging in Africa longitudinal study HIV, hypertension, diabetes, dyslipidemia, angina, depression, PTSD, alcohol dependence

George et al., 2019 [32]	The association between a detectable HIV viral load and non-communicable diseases comorbidity in HIV positive adults on antiretroviral therapy in Western Cape, South Africa	Khayelitsh, Cape Town, South Africa	Cross-sectional study	HIV-infected adults (n = 330) on ART who attend the HIV clinic within a primary health centre HIV, hypertension, diabetes, chronic respiratory disease, epilepsy

Iwelunmor et al., 2019 [33]	Capabilities, opportunities and motivations for integrating evidence-based strategy for hypertension control into HIV clinics in Southwest Nigeria	Lagos, Nigeria	Concurrent Quan-Qual study (Structured questionnaires followed by Stakeholder meetings)	HIV clinics (n = 29) HIV, hypertension

Kwarisiima et al., 2019 [34]	Hypertension control in integrated HIV and chronic disease clinics in Uganda in the SEARCH study	Rural Uganda	Cluster RCT	Residents (n = 34,704) of ten communities in rural Uganda HIV, hypertension

Ojo et al., 2019 [35]	Feasibility of integrated, multilevel care for cardiovascular diseases (CVD) and HIV in low and middle-income countries (LMICs): A scoping review	Sub-Saharan Africa, Southeast Asia, South America	Scoping review	Studies (n = 20): articles (n = 18), conference abstracts (n = 3) HIV, cardiovascular diseases

Ansbro et al, 2018 [36]	Evaluation of NCD service integrated into a general OPD and HIV service in Matsapha, Eswatini, 2017	Swaziland	Retrospective descriptive cohort study	Patients (n = 895) enrolled at Matsapha Comprehensive Care Clinic Cardiovascular disease, hypertension, diabetes mellitus, chronic respiratory disease

Ekrikpo et al., 2018 [37]	Prevalence and correlates of traditional risk factors for cardiovascular disease in a Nigerian ART-naive HIV population: a cross-sectional study	Southern Nigeria	Cross-sectional study	Antiretrovirals-naïve patients (n = 12,167) initiating care at the University of Uyo Teaching Hospital HIV clinic HIV, hypertension, DM, obesity, dyslipaedemia

Golovaty et al., 2018 [38]	Cost of Integrating Noncommunicable Disease Screening Into Home-Based HIV Testing and Counselling in South Africa	KwaZulu-Natal, South Africa	Cross-sectional costing analysis	People (n = 570) who received integrated HIV-NCD testing, counselling and referral to care in January 2015 HIV, diabetes, hypertension, hypercholesterolemia, obesity, depression

Haldane et al., 2018 [39]	Integrating cardiovascular diseases, hypertension, and diabetes with HIV services: a systematic review	Sub-Saharan Africa, Cambodia, UK, USA	Systematic review	Articles (n = 14) representing 17 studies HIV/AIDS, CVDs, hypertension, diabetes, cerebrovascular diseases

Juma et al., 2018 [40]	From HIV prevention to non-communicable disease health promotion efforts in sub-Saharan Africa: A Narrative Review	SSA	Narrative literature review	SSA-based studies (n = 20) on NCDs and dual HIV/NCD health promotion interventions HIV, cardiovascular disease, type 2 diabetes, cervical cancer, depression

Matanje et al., 2018 [41]	Opportunities and challenges for evidence-informed HIV-noncommunicable disease integrated care policies and programs: lessons from Malawi, South Africa, Swaziland and Kenya	Malawi, South Africa, Swaziland, Kenya	Cross-sectional analysis	Current policies and programs relating to HIV/NCD integration HIV, NCDs

Njuguna et al, 2018 [19]	Models of integration of HIV and noncommunicable disease care in sub-Saharan Africa: lessons learned and evidence gaps	SSA	Narrative review of the literature	N = 5 case studies HIV, non-communicable diseases

Nuche-Berenguer and Kupfer, 2018 [42]	Readiness of Sub-Saharan Africa Healthcare Systems for the New Pandemic, Diabetes: A Systematic Review	SSA	Systematic review	Articles (n = 55) on Embase, Scopus, PubMed Diabetes, HIV

Patel et al., 2018 [43]	Noncommunicable diseases among HIV-infected persons in low income and middle-income countries: a systematic review and meta-analysis	Low- and middle-income countries, with a focus on SSA	Systematic review and meta-analysis	Peer-reviewed literature (n = 141); 57 included in qualitative analysis HIV, CVD, cervical cancer, depression, diabetes

Pfaff et al., 2018 [44]	Early experiences integrating hypertension and diabetes screening and treatment in a human immunodeficiency virus clinic in Malawi	Zomba District, Malawi	Pilot study	Adults HIV patients (n = 6036) HIV, diabetes, hypertension

Rawat et al., 2018 [45]	Integrated HIV-care into primary care clinics and the influence on diabetes and hypertension care: an interrupted time series analysis in Free State, South Africa over four years	Free State, South Africa	Quasi-experimental design	Data from primary health care clinics (n = 131) with a catchment population of 1.5 million HIV, diabetes, hypertension

Ameh et al, 2017 [46]	Effectiveness of an Integrated Approach to HIV and Hypertension Care in Rural South Africa: Controlled Interrupted Time-Series Analysis	Bushbuckridge municipality, Mpumalanga, South Africa	Controlled interrupted time-series study	Patients (n = 443) from the primary healthcare facilities in Buckbuckridge HIV, hypertension

Mosha et al., 2017 [47]		North West Tanzania	Community-based cross-sectional study	Adults (n = 9,678) in Magu District in 2013 HIV, hypertension, diabetes, and other chronic diseases

Pfaff et al., 2017 [48]	You can treat my HIV – But can you treat my blood pressure? Availability of integrated HIV and noncommunicable disease care in northern Malawi	Rural Northern Malawi	Sequential mixed methods: Cross-sectional survey and semi-structured interviews	Health centres (n = 25) and hospitals (n = 5); NCD coordinators (n = 3) HIV, hypertension, diabetes

Shankalala et al., 2017 [49]	Risk factors for impaired fasting glucose or diabetes among HIV infected patients on ART in the Copperbelt Province of Zambia	Copperbelt Province, Zambia	Cross-sectional study	HIV/AIDs patients (n = 270) on Combined Antiretroviral Treatment (cART) for more than 2 years HIV, diabetes

Divala et al, 2016	The burden of hypertension, diabetes mellitus, and cardiovascular risk factors among adult Malawians in HIV care: consequences for integrated services	Malawi	Cross-sectional study	Patients (n = 952) at the HIV clinics of Zomba Central Hospital HIV, hypertension, diabetes

Fairall et al., 2016 [50]	Educational Outreach with an Integrated Clinical Tool for Nurse-Led Noncommunicable Chronic Disease Management in Primary Care in South Africa: A Pragmatic Cluster Randomised Controlled Trial	Rural and Urban South Africa	Cluster randomized controlled trial	Patients (n = 4,393) of public sector primary healthcare clinics (n = 38) Hypertension, diabetes, chronic respiratory disease, depression

Rachlis et al., 2016 [51]	Identifying common barriers and facilitators to linkage and retention in chronic disease care in western Kenya	Western Kenya	Exploratory qualitative study (in-depth interviews and focus group discussions)	Participants (n = 235) sampled from 3 AMPATH clinics: Individuals living with HIV (n = 50), TB (n = 39), hypertension (n = 21); caregivers (n = 24), community leaders (n = 10), healthcare providers (n = 62) HIV, tuberculosis (TB), hypertension

Some et al., 2016 [52]	Task Shifting the Management of Non- Communicable Diseases to Nurses in Kibera, Kenya: Does It Work?	Kenya, Kibera	Descriptive, retrospective review	Consultations (n = 725) by nurses with patients (n = 616) in 2 integrated primary health care facilities Hypertension, T2DM, epilepsy, asthma, sickle cell disease

Edwards et al, 2015 [53]	HIV with non-communicable diseases in primary care in Kibera, Nairobi, Kenya: characteristics and outcomes 2010–2013	Kibera, Nairobi, Kenya	Retrospective descriptive cohort study	Patients (n = 2206) >14 years with hypertension and/or diabetes registered in the chronic diseases clinic from Jan 2010- June 2013 HIV, hypertension, diabetes

Haregu et al., 2015 [54]	Integration of HIV/AIDS and noncommunicable diseases in developing countries: rationale, policies and models	Developing countries in SSA region and Cambodia	Interpretative qualitative synthesis	References from PubMed and general search engines and websites of WHO, UNAIDS and the NCD alliance

				HIV/AIDS, CVDs, cancers, diabetes, COPD

Khabala et al, 2015 [55]	Medication Adherence Clubs: a potential solution to managing large numbers of stable patients with multiple chronic diseases in informal settlements	Kibera, Nairobi, Kenya	Retrospective, descriptive study	Patients (n = 1432) from 47 Medication Adherence Clubs (MACs) HIV, hypertension, diabetes,

Mahomed and Asmall, 2015 [56]	Development and implementation of an integrated chronic disease model in South Africa: lessons in the management of change through improving the quality of clinical practice	South Africa	Pre-post study	Primary health care facilities (n = 42)

Joshi et al., 2014 [57]	Task Shifting for Non-Communicable Disease Management in Low and Middle Income Countries – A Systematic Review	Low and middle income countries e.g. Ethiopia, Kenya, Tanzania, Zimbabwe	Systematic review	RCTs (n = 7) and observational studies (n = 15) Hypertension, diabetes, CVD, mental health, neurological conditions, COPD, cancer

Govindasamy et al, 2013 [58]	Linkage to HIV, TB and Non-Communicable Disease Care from a Mobile Testing Unit in Cape Town, South Africa	Cape Town, South Africa	Observational cohort study	Clients (n = 9806) screened at mobile testing unit HIV, TB, diabetes, hypertension,

Chamie et al, 2012 [59]	Leveraging rapid community-based HIV testing campaigns for noncommunicable diseases in rural Uganda	Uganda	Feasibility study Controlled before-after design	Residents (n = 6300) of the rural Ugandan parish HIV, malaria, TB, hypertension, diabetes Community members (n = 4343)

Rabkin et al., 2012 [18]	Strengthening Health Systems for Chronic Care: Leveraging HIV Programs to Support Diabetes Services in Ethiopia and Swaziland	Swaziland, Ethiopia	2 feasibility/proof-of-concept studies	Patients (n = 100 in Swaziland and n = 260 in Ethiopia) with DM whose charts were randomly reviewed HIV, diabetes mellitus

Van Olmen et al., 2012 [60]	Management of Chronic Diseases in Sub-Saharan Africa: Cross-Fertilisation between HIV/AIDS and Diabetes Care	SSA	Literature review	Patients with DM and HIV/AIDS Diabetes mellitus type 2 and HIV/AIDS.

Levitt et al., 2011 [61]	Chronic noncommunicable diseases and HIV-AIDS on a collision course: relevance for health care delivery, particularly in low-resource settings—insights from South Africa	South Africa	Narrative review	HIV, NCDs

Lekoubou et al., 2010 [62]	Hypertension, Diabetes Mellitus and Task Shifting in Their Management in Sub-Saharan Africa	SSA South Africa, Cameroon	Narrative literature review	Articles (n = 5) Hypertension, diabetes

Maher et al., 2010 [63]	Health transition in Africa: practical policy proposals for primary care	SSA	Narrative review	Patients with co-morbid communicable diseases and NCDs Communicable (CDs) and non-communicable diseases (NCDs)

* PLHIV – People living with HIV. * LMIC – Low-and-middle income countries.
